# Laparoscopic transabdominal preperitoneal repair for a patient with Laugier’s and inguinal hernia

**DOI:** 10.1186/s40792-024-02017-2

**Published:** 2024-09-11

**Authors:** Masaaki Yamamoto, Atsushi Takeno, Reishi Toshiyama, Shinji Tokuyama, Kenji Kawai, Yusuke Takahashi, Kenji Sakai, Naoki Hama, Takeshi Kato, Motohiro Hirao

**Affiliations:** Department of Surgery, NHO Osaka National Hospital, 2-1-14 Hoenzaka, Chuo-Ku, Osaka, 540-0006 Japan

**Keywords:** Laugier’s hernia, Inguinal hernia, Femoral hernia, Laparoscopic transabdominal preperitoneal repair, Lacunar ligament, Gimbernat’s ligament

## Abstract

**Background:**

Laugier’s hernia is a very rare atypical femoral hernia and is challenging to diagnose preoperatively. Herein, we report a rare case of inguinal and Laugier’s hernias treated with laparoscopic transabdominal preperitoneal repair.

**Case presentation:**

A 63-year-old man was admitted to our hospital with right groin swelling for 4 years. Computed tomography revealed an indirect inguinal hernia with protrusion of the small intestine. The preoperative diagnosis was right indirect inguinal hernia; Laugier’s hernia was unknown. The patient underwent laparoscopic transabdominal preperitoneal repair. During the surgery, part of the perivesical adipose tissue penetrated the lacunar ligament. It was located medial to the typical site of a femoral hernia. Thus, Laugier's hernia was diagnosed. Finally, laparoscopic transabdominal preperitoneal repair was performed for Laugier's hernia and inguinal hernia. The postoperative course was good, without recurrence.

**Conclusions:**

To our knowledge, this is the first reported case of inguinal and Laugier’s hernia treated with laparoscopic transabdominal preperitoneal repair. Surgeons should be mindful that inguinal hernias can occur concurrently with other types of hernias, such as femoral hernias, including atypical variants like Laugier's hernia. Additionally, they should actively consider laparoscopic approaches such as transabdominal preperitoneal for femoral hernias. These approaches are beneficial for precise diagnosis, confirming the presence of other hernias, and simultaneously treating all coexisting inguinal hernias.

## Background

Femoral hernias account for approximately 4% of all groin hernias [1–4]. Moreover, as an atypical type of femoral hernia, although very rare, several subtypes of femoral hernias occur in different atypical locations, developing through the lacunar ligament (Laugier’s hernia), pectineal fascia (Cloquet’s hernia), or in relation to the femoral vessels; lateral (Hesselbach’s hernia), prevascular (Velpeuau’s hernia or Teale’s hernia), or retrovascular (Serafini’s hernia). Among these, Laugier’s hernia is very rare, with only a few reports [5–8]. Herein, we report a rare case of inguinal and Laugier’s hernias treated with laparoscopic transabdominal preperitoneal repair (TAPP).

## Case presentation

A 63-year-old man was admitted to our hospital with right groin swelling for 4 years. When the patient was in an upright position, a bulge was observed in the right groin, which naturally reduced when the patient was in a supine position. A computed tomography (CT) scan revealed a right indirect inguinal hernia with protrusion of the small intestine. The preoperative diagnosis was right indirect inguinal hernia, and Laugier’s hernia was unknown.

**Fig. 1:**
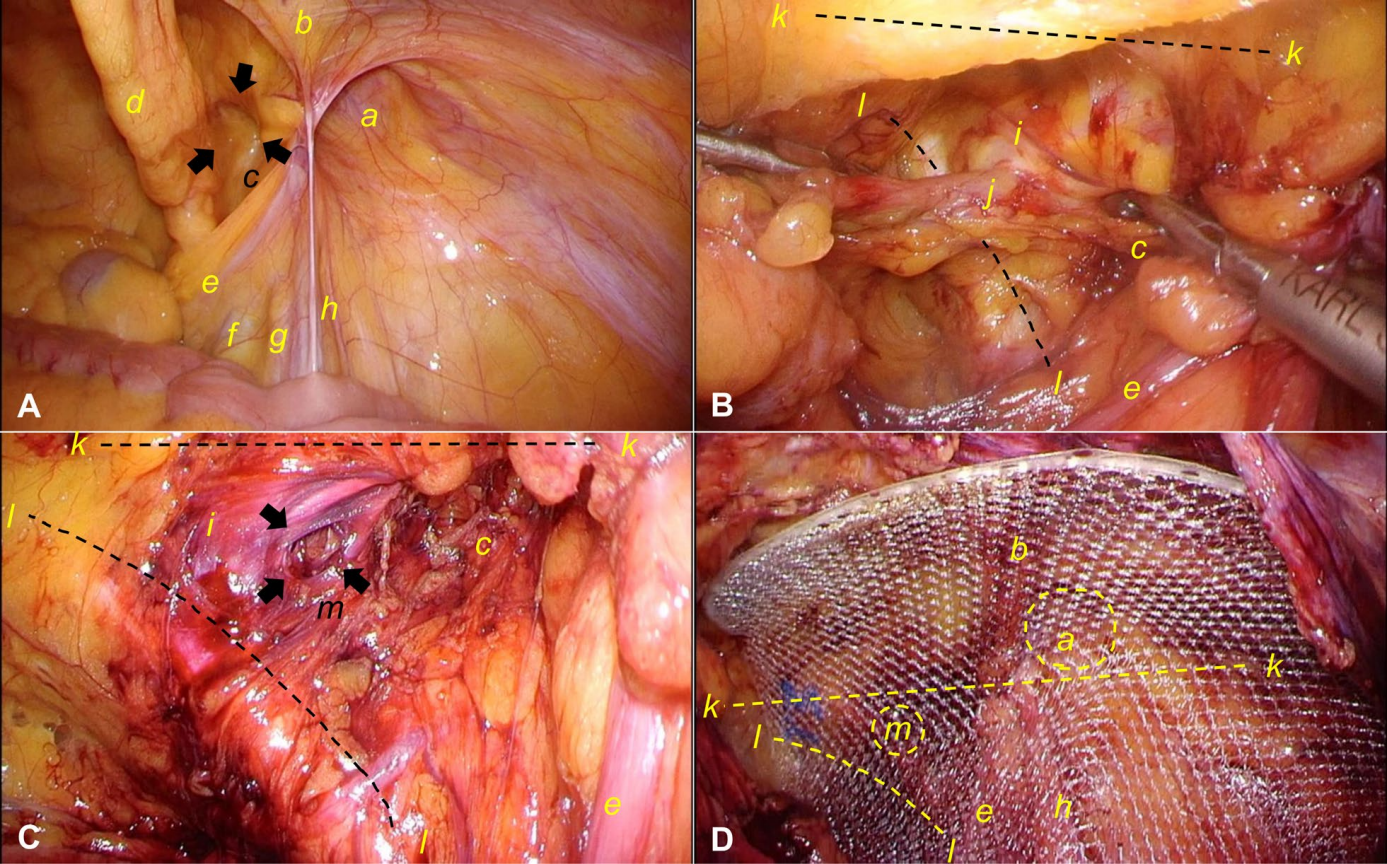
Laparoscopic view of the right Laugier’s hernia. **a** Right indirect inguinal hernia. The right femoral ring is slightly depressed. **b** Part of the perivesical adipose tissue penetrates the lacunar ligament. **c** When the adipose tissue is pulled out, an approximately 8 mm hole is found in the lacunar ligament. It is located medial to the typical site of a femoral hernia, and a diagnosis of Laugier's hernia is made. **d** Polypropylene mesh is inserted into the preperitoneal space covering the myopectineal orifice, including Laugier’s hernia orifice and indirect inguinal hernia orifice: *a* right indirect inguinal hernia orifice; *b* right inferior epigastric vessels; *c* femoral canal (arrows); *d* right medial umbilical fold; *e* right spermatic cord; *f* right external iliac vein; *g* right external iliac artery; *h* right testicular vessels; *i* lacunar ligament; *j* perivesical adipose tissue; *k* right iliopubic tract; *l* Cooper’s ligament; *m* Laugier’s hernia orifice (arrows)

The patient underwent TAPP. After the induction of general anesthesia, the patient was placed in the supine position, and a 2-cm incision was made at the umbilicus. A 12-mm port was placed for the camera. After placing the 12-mm port, CO_2_ was insufflated to a pressure of 10 mmHg, and using a 30° laparoscope, two 5-mm ports were placed at 3–4-finger width at the left caudal and the right of the umbilicus. After the three ports were placed, the patient was placed in head-down and right-up tilt positions. Laparoscopy revealed that the right indirect inguinal hernia and the right femoral ring were slightly depressed (Fig. 1a). A peritoneal incision was made on the lateral side of the hernial orifice. The inferior epigastric artery and vein, vas deferens, and testicular artery and vein were identified. As the dissection proceeded toward the prevesical space, part of the perivesical adipose tissue penetrated the lacunar ligament (Fig. 1b). When the adipose tissue was removed, an approximately 8 mm hole was observed in the lacunar ligament (Fig. 1c). It was located medial to the typical site of a femoral hernia and a diagnosis of Laugier's hernia was made. Hence, the patient was diagnosed with a right indirect inguinal hernia and Laugier's hernia.

Subsequently, dissection was performed just beyond the midline of the pubic tubercle and continued until the mesh was adequately deployed. Horizontally, a 16 cm long dissection was performed from the pubic tubercle along the iliopubic tract toward the right anterior superior iliac spine; approximately an 11 cm long dissection was performed vertically from the periphery of the inferior abdominal wall artery and vein to the dissected part of the testicular artery and vein. A 10.8 × 16.0 cm polypropylene mesh (3DMax™ Mesh, Large Right, Bard) was inserted into the preperitoneal space covering the myopectineal orifice, which is an area surrounded by the rectus abdominis muscle, the transversus abdominis aponeurotic arch, Cooper's ligament, and the iliac fascia, and which consists of the Hesselbach’s triangle, internal inguinal ring, and femoral ring (Fig. 1d). After fixing the outer edge of the mesh by tacking, the peritoneum was closed with 3–0 absorbable monofilament. Subsequently, the skin was closed with 4–0 absorbable monofilament dermal sutures to complete the surgery. The patient was discharged 3 days after surgery without complications. No recurrence was observed at the 1-month follow-up.

## Discussion

To our knowledge, this is the first report of a patient with inguinal and Laugier’s hernia who underwent TAPP. Laugier’s hernia was first reported as a herniation of the peritoneal sac through the lacunar ligament (Gimbernat’s ligament) by Henrie de Laugier in 1833 and is classified as an atypical femoral hernia [9]. Although femoral hernias are more frequent in women than in men, the sex ratio of Laugier’s hernia is unknown. Accurately diagnosing Laugier’s hernia before surgery is challenging because of its rarity and pathway similar to that of a typical femoral hernia. The limited number of reports on Laugier’s hernia may be because of low awareness of the disease and misdiagnosis as a typical femoral hernia. Additionally, Laugier's hernia is challenging to diagnose via traditional open surgery, which has a limited surgical field of view. This may explain the limited reports on it. However, with the recent widespread implementation of the laparoscopic approach, diagnosis has become easier owing to good surgical field of view, which is an advantage of this approach. Thus, the number of reports may increase in the future. In this case, swelling at the femoral site was not observed, and CT was unable to detect a Laugier’s hernia in the patient before surgery. One of the causes of femoral hernia may be chronically increased abdominal pressure, such as chronic coughing, as observed in other inguinal hernias. Therefore, Laugier’s hernia may have been caused by the same reason. Another cause, similar to that for other inguinal hernias, may be poor collagen quality. Although most Laugier’s hernias are asymptomatic, femoral swelling can occur if the herniated contents are large similar to typical femoral hernias [5]. The contents of Laugier’s hernia are considered to be mostly preperitoneal fat. If the preperitoneal fat becomes incarcerated, pain may be observed in patients with strangulated umbilical hernias [10]. Moreover, bladder hernia may occur owing to traction by the incarcerated preperitoneal fat. Therefore, we believe that carefully and thoroughly removing or dissecting the trapped fat tissue from the lacuna ligament without damaging the bladder should be recommended. In the present case, preoperative CT and intraoperative findings revealed no bladder hernia.

The treatment principle for Laugier’s hernia is the reduction of the hernia contents and the covering of the hernia orifice formed in the lacunar ligament with a mesh. Laparoscopic surgery, including TAPP or totally extraperitoneal repair (TEP), is advantageous over open surgery because the presence of a Laugier’s hernia or other hernias can be detected simultaneously, more easily and clearly, and can be easily treated. Ates et al*.* first reported Laugier’s hernia treated with TEP [5]. In that case, the patient had groin swelling and pain and was diagnosed as a typical femoral hernia before surgery. However, the patient was re-diagnosed as having Laugier’s hernia during TEP. Therefore, they reported that it was difficult to diagnose Laugier’s hernia before surgery and that the laparoscopic approach should be chosen instead of a conventional approach. Therefore, a laparoscopic approach should be considered for patients with femoral hernias. Recent International Hernia guidelines recommend a laparoscopic approach with the insertion of a pre-peritoneal mesh as the standard for primary femoral hernia repair [11]. According to the guidelines, this recommendation is partly based on the clinical inaccuracy in distinguishing an inguinal hernia from a femoral hernia.

## Conclusions

Surgeons should consider that inguinal hernias may coexist with other hernias, including femoral hernias, especially atypical femoral hernias such as Laugier’s hernia, as in this case. Furthermore, laparoscopic approaches such as TAPP should be actively considered for femoral hernias because they are useful for accurate diagnosis, confirmation of the presence of other hernias, and simultaneous treatment of all coexisting inguinal hernias.

## Data Availability

The data are not available for public access because of patient privacy concerns but are available from the corresponding author upon reasonable request.

## Abbreviations

TAPP: Laparoscopic transabdominal preperitoneal repair
CT: Computed tomography
TEP: Totally extraperitoneal repair
